# IL-15 deficient Tax mice reveal a role for IL-1α in tumor immunity

**DOI:** 10.1186/1742-4690-11-S1-O11

**Published:** 2014-01-07

**Authors:** Daniel A Rauch, John C Harding, Lee Ratner

**Affiliations:** 1 Department of Medicine, Washington University School of Medicine, St. Louis, Missouri, USA

## 

IL-15 is recognized as a promising candidate for tumor immunotherapy and has been described as both a promoter of cancer and a promoter of anti-cancer immunity. IL-15 was discovered in cells transformed by HTLV-1, the etiologic agent of adult T cell leukemia / lymphoma (ATL) and the human retrovirus that carries the Tax oncogene. We have developed the TAX-LUC mouse model of ATL in which Tax expression drives both malignant transformation and luciferase expression, enabling non-invasive imaging of tumorigenesis in real time. To identify the role of IL-15 in spontaneous development of lymphoma in vivo, an IL-15-/- TAX-LUC strain was developed and examined. The absence of IL-15 resulted in aggressive tumor growth and accelerated mortality and demonstrated that IL-15 was not required for Tax-mediated lymphoma but was essential for anti-tumor immunity. Further analysis revealed a unique transcriptional profile in tumor cells that arise in the absence of IL-15 that included a significant increase in the expression of IL-1α and IL-1α-regulated cytokines. Moreover, anti-IL-1α antibodies and an IL-1 receptor antagonist (Anakinra) were used to interrogate the potential of IL-1α targeted therapies in this model. Taken together, these findings identify IL-15 and IL-1α as therapeutic targets in lymphoma.

